# Gut Bacterial Communities of *Dendroctonus valens* and *D. mexicanus* (Curculionidae: Scolytinae): A Metagenomic Analysis across Different Geographical Locations in Mexico

**DOI:** 10.3390/ijms19092578

**Published:** 2018-08-30

**Authors:** Juan Alfredo Hernández-García, Roman Gonzalez-Escobedo, Carlos Iván Briones-Roblero, Claudia Cano-Ramírez, Flor N. Rivera-Orduña, Gerardo Zúñiga

**Affiliations:** 1 Posgrado en Ciencias Quimicobiológicas, Escuela Nacional de Ciencias Biológicas, Instituto Politécnico Nacional, Mexico City, CP 11340, Mexico; freddyhardcore@hotmail.com (J.A.H.-G.); romanglze@hotmail.com (R.G.-E.); 2 Laboratorio de Variación Biológica y Evolución, Departamento de Zoología, Escuela, Nacional de Ciencias Biológicas, Instituto Politécnico Nacional, Prolongación de Carpio y Plan de Ayala s/n, Delegación Miguel Hidalgo, Mexico City, CP 11340, Mexico; cabriones13@gmail.com (C.I.B.-R.); clacanram@yahoo.com.mx (C.C.-R.); 3 Laboratorio de Ecología Microbiana, Departamento de Microbiología, Escuela Nacional de Ciencias Biológicas, Instituto Politécnico Nacional, Prolongación de Carpio y Plan de Ayala s/n, Delegación Miguel Hidalgo, Mexico City, CP 11340, Mexico; flor_1413@hotmail.com

**Keywords:** gut bacteriome, core bacteriome, geographical variation

## Abstract

*Dendroctonus* bark beetles are a worldwide significant pest of conifers. This genus comprises 20 species found in North and Central America, and Eurasia. Several studies have documented the microbiota associated with these bark beetles, but little is known regarding how the gut bacterial communities change across host range distribution. We use pyrosequencing to characterize the gut bacterial communities associated with six populations of *Dendroctonus valens* and *D. mexicanus* each across Mexico, determine the core bacteriome of both insects and infer the metabolic pathways of these communities with Phylogenetic Investigation of Communities by Reconstruction of Unobserved States (PICRUSt) to evaluate whether these routes are conserved across geographical locations. Our results show that the β-diversity with UniFrac unweighted varies among locations of both bark beetles mainly due to absence/presence of some rare taxa. No association is found between the pairwise phylogenetic distance of bacterial communities and geographic distance. A strict intraspecific core bacteriome is determined for each bark beetle species, but these cores are different in composition and abundance. However, both bark beetles share the interspecific core bacteriome recorded previously for the *Dendroctonus* genus consisting of *Enterobacter*, *Pantoea*, *Providencia*, *Pseudomonas*, *Rahnella*, and *Serratia*. The predictions of metabolic pathways are the same in the different localities, suggesting that they are conserved through the geographical locations.

## 1. Introduction

*Dendroctonus* Erichson (Curculionidae: Scolytinae) bark beetles are not only a natural inhabitant of coniferous forests but also a major pest during the periodic outbreaks in pine forests from North and Central America [1,2]. Many studies have revealed that these insects are associated with facultative microbes, and it has been demonstrated through in vitro tests, the amplification of specific gene, and sequencing technologies that some of the microbes provide benefits to bark beetles favoring their adaptation and reproductive success [3,4,5,6]. Bacteria can aid in the digestion of plant structural carbohydrates [7,8,9], nitrogen fixation and recycling [10,11,12,13], terpene detoxification [14,15,16], pheromone production [17], protection against antagonistic fungi [18,19], and mediation of carbohydrate consumption and growth of associated fungi [20].

Several studies using conventional techniques and massive parallel sequencing technologies have allowed the determination of the bacterial community structure associated with the inside (e.g., gut and mycangium) and the outside (e.g., mouthparts and cuticle) of the bark beetles body [7,12,21,22,23,24,25,26,27,28], galleries [29], and specific developmental stages of some species [30,31]. In addition, the presence of two core bacteriomes, one strict (composed by *Enterobacter*, *Serratia*, *Rahnella*, *Pantoea*, *Raoultella*, and *Pseudomonas*) and another relaxed (constituted of the strict core plus *Acinetobacter*, *Propionibacterium*, *Providencia*, *Stenotrophomonas*, *Erwinia*, *Kluyvera*, *Paenibacillus*, and *Ralstonia*) in the gut has been recently determined in different *Dendroctonus* species [28], which suggests that some of these taxa probably support basic metabolic processes that benefit these bark beetles.

Based on this evidence, and notwithstanding that the *Dendroctonus* bark beetles’ gut presumably is a heterogeneous yet limiting habitat, it is reasonable to hypothesize that the strict core bacteriome of these insects should be stable across the distribution range of species, despite the variation in the richness and evenness of the bacterial community across the geographical space. Three pieces of evidence support this idea: (1) The low bacterial diversity observed in many studies performed on bark beetles [26,27,30], and other insects with a similar diet as pine weevils [32], and cerambycids [33] with respect to those found in other phytophagous insects [34,35,36,37]; (2) The dominance of some taxa of the core bacteriome and the presence/absence of rare taxa [28]; and (3) The higher diversity of endophytic bacteria found in the pine species phloem, a substrate on which bark beetles feed, with respect to the diversity found in their gut [38].

There were two aims in this study: (1) To analyze the gut bacterial community of two bark beetle species, *Dendroctonus valens* LeConte and *D*. *mexicanus* Hopkins, across six different geographic localities using 16S rRNA pyrosequencing. The first species has a wide distribution in North and Central America, where it is a parasite of stumps or trees of more than 40 pine species [39,40], while the second species is found mainly in Mexico and it is an aggressive species that attacks and kills approximately 20 pine species in its distribution range [39]; (2) To infer the metabolic pathways of the entire bacterial community in each locality of these bark beetles with the Phylogenetic Investigation of Communities by Reconstruction of Unobserved States (PICRUSt) to evaluate whether these metabolic processes are conserved across the geographical space studied. Although we have some idea of the specific metabolic capacities of some bacterial species found in the bark beetles’ gut, this inference is the first approximation to know the integral functional role of the entire community, as well as to explore whether the predicted metabolic pathways are maintained across the geographic space.

## 2. Results

### 2.1. Taxonomic Analysis of Gut Bacterial Communities

**Figure 1:**
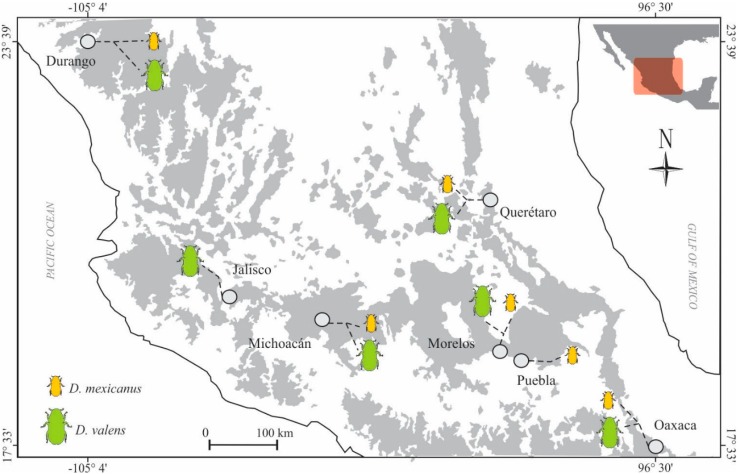
Locations studied of the *Dendroctonus valens* (green) and *D. mexicanus* (orange). Grey dot = sampled localities; dot line = collected species.

A total of 155,324 and 297,425 raw reads were obtained from six localities (Figure 1, Table S1) and replicates for *Dendroctonus valens* and *D*. *mexicanus*, respectively. After the quality control, 86,349 reads for *D*. *valens* and 101,512 reads for *D*. *mexicanus* remained for subsequent analyses. The Good’s coverage index values were greater than 97% in all locations of both bark beetles (Table 1).

**Figure 2:**
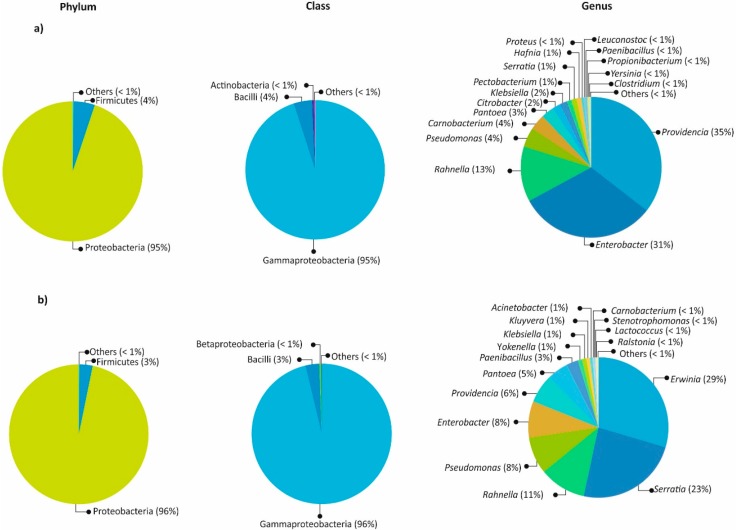
Relative abundances of the most abundant taxa of the gut at phylum, class, family, and genus levels: (**a**) *Dendroctonus valens*, (**b**) *D. mexicanus*.

**Figure 3:**
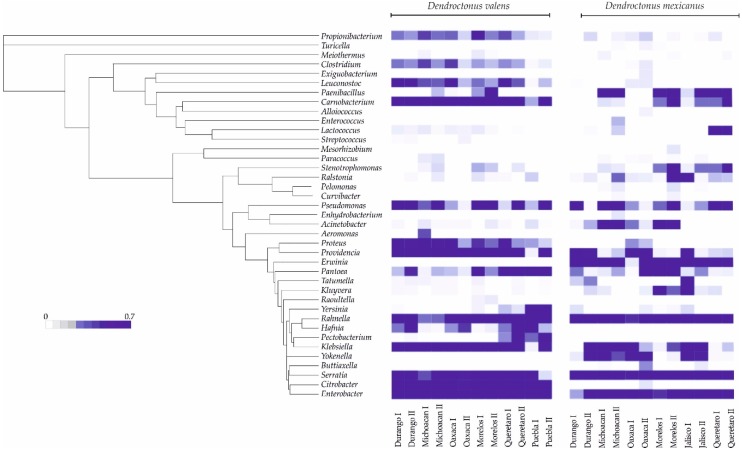
Heatmap of representative sequences of operational taxonomic units (OTUs) of *Dendroctonus valens* and *D. mexicanus* in different localities. The range of colors indicates the OTUs relative abundance for each sample; dark colors indicate higher abundance and light ones lower abundance.

The gut bacterial communities were similar in composition and abundance among the locations of both bark beetles. The most abundant phylum in both species was Proteobacteria, followed by Firmicutes, Actinobacteria, and Deinococcus-Thermus (Figure 1). At the genus level, the most abundant were in *Dendroctonus valens*—*Providencia* (35%), *Enterobacter* (31%), *Rahnella* (13%), *Pseudomonas* (4%), *Carnobacterium* (4%), *Pantoea* (3%), *Citrobacter* (2%), *Klebsiella* (2%), *Pectobacterium* (1%), *Serratia* (1%), and *Hafnia* (1%)—while in *D*. *mexicanus*, they were *Erwinia* (29%), *Serratia* (23%), *Rahnella* (11%), *Pseudomonas* (8%), *Enterobacter* (8%), *Providencia* (6%), *Pantoea* (5%), *Paenibacillus* (3%), *Klebsiella* (1%), *Kluyvera* (1%), *Acinetobacter* (1%), and *Yokenella* (1%). Other genera with <1.0% relative abundances were found in both species (Figure 2 and Figure 3).

### 2.2. Bacterial Community Diversity

The operational taxonomic units (OTUs) richness, evenness, and overall diversity of the bacteria were similar among localities in both bark beetles, except for the Michoacan locality in *Dendroctonus valens*. An average of 127 and 84.5 of the observed OTUs was recorded across locations for *D*. *valens* and *D*. *mexicanus*, respectively. The higher number of OTUs was detected in Morelos I (177) and Oaxaca I (102) for *D*. *valens* and *D*. *mexicanus*, respectively (Table 1). 

The values of species richness (Chao1) were not statistically different among the locations of *Dendroctonus mexicanus* (*F* Welch test: *F*_Chao1_ = 1.7, *p* = 0.35), but whether among *D. valens* localities (*F* Welch test: *F*_Chao1_ = 130.8, *p* = 0.003) being only different the Michoacan location (Michoacan vs. Oaxaca; *p* = 0.02). The values of the Shannon diversity index varied from 3.4 to 4.9 among the *D*. *valens* locations and from 2.5 to 4.0 in those of *D*. *mexicanus*. This index did not show differences among the *D*. *mexicanus* populations (*F* Welch test: *F*_Shannon_ = 0.17, *p* = 0.95), but whether among *D*. *valens* locations (*F* Welch test: *F*_Shannon_ = 15.9, *p* = 0.03) where the Michoacan locality was different to the Queretaro locality (*p* = 0.009). The values of the phylogenetic diversity (PD) index were not statistically different among locations of *D*. *mexicanus* (*F* Welch test: *F*_PD_ = 0.84, *p* = 0.6) but whether among the *D*. *valens* locations (*F*_PD_ = 16.9, *p* = 0.03) where the Jalisco locality was different to the Morelos (*p* = 0.01). Lastly, the values of the Simpson (S) index did not show statistically significant differences among locations of the *D*. *mexicanus* (*F* Welch test: *F*_S_ = 1.5, *p* = 0.41) but whether among the *D*. *valens* locations (*F* Welch test: *F*_S_ = 13.7, *p* = 0.04) where the Michoacan locality was different from the Queretaro (*p* = 0.04; Table 1).

**Figure 4:**
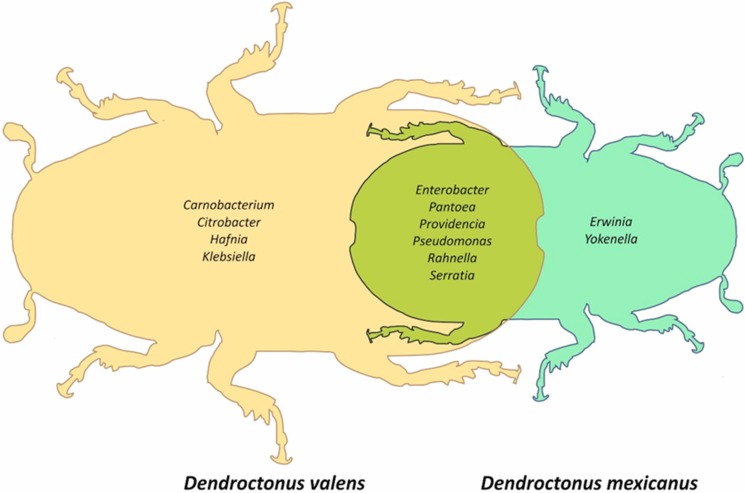
Venn diagram representing unique and shared taxa present in all localities between *Dendroctonus valens* and *D. mexicanus*.

The *Dendroctonus valens* localities shared a strict core bacteriome (Table S4) constituted by 10 bacterial genera, namely *Citrobacter*, *Carnobacterium*, *Enterobacter*, *Hafnia*, *Klebsiella*, *Pantoea*, *Providencia*, *Pseudomonas*, *Rahnella*, and *Serratia*, whose sequences represented 93.8% of the total reads. On the other hand, the *D*. *mexicanus* localities shared a strict core bacteriome (Table S4) constituted by eight taxa, namely *Enterobacter*, *Erwinia*, *Pantoea*, *Pseudomonas*, *Providencia*, *Rahnella*, *Serratia*, and *Yokenella*, representing 94% of the total reads. The Venn diagram built for both species based on all localities showed that only six bacterial genera—*Enterobacter*, *Pantoea*, *Providencia*, *Pseudomonas*, *Rahnella*, and *Serratia*—were shared among these bark beetles (Figure 4).

**Figure 5:**
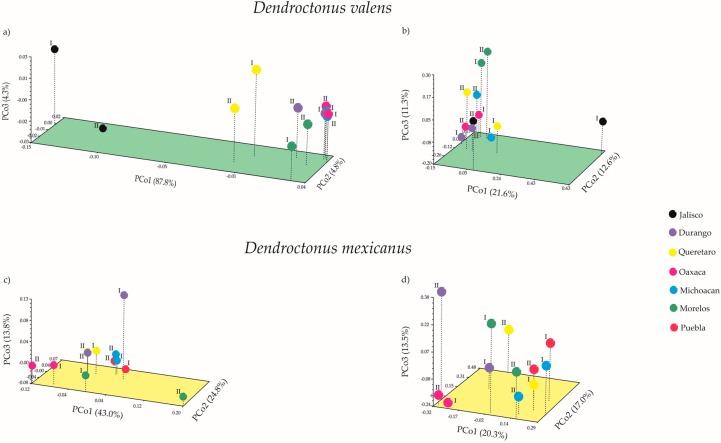
Principal coordinates analysis (PCoA) of bacterial communities of *Dendroctonus valens* (**a**,**b**) and *D. mexicanus* (**c**,**d**) using weighted and unweighted UniFrac distances, respectively. I = replicate one; II = replicate two.

The first three coordinates of the Principal Coordinates Analysis (PCoA) in *Dendroctonus* *valens* using weighted UniFrac (wU) and unweighted UniFrac (uwU) distances explained 96.9% (PCo1—87.8%; PCo2—4.8%; PCo3—4.3%) and 45.5% (PCo1—21.6%; PCo2—12.6%; PCo3—11.3%) of the total variation, respectively (Figure 5a,b). In *D*. *mexicanus* the PCoA (wU) explained 81.6% (PCo1—43.0%; PCo2—24.8%; PCo3—13.8%) and the PCoA (uwU) 50.8% (PCo1—20.3%; PCo2—17.0%; PCo3—13.5%) of the total variation (Figure 5c,d). Significant differences were found in the bacterial β-diversity among the communities of different localities for both bark beetles using unweighted UniFrac with the Monte Carlo test (Table S2, *p* < 0.05). These differences were mainly due to the presence or absence of rare taxa (e.g., *Turicella*, *Meiothermus*, *Exiguobacterium*, *Curvibacter*, among others), which have a relative frequency lower than 1% (Figure 3, light colors). Meanwhile, no significant differences were found among the bacterial communities of both *Dendroctonus* species when the relative abundance was weighted (Table S2, *p* > 0.05). These significant differences in β-diversity of both *Dendroctonus* species were confirmed with the Adonis test using the pairwise Bray-Curtis dissimilarity matrices (*D*. *valens p* = 0.001, *R*^2^ = 0.85; *D*. *mexicanus p* = 0.006, *R*^2^ = 0.71).

Lastly, the correlation between the pairwise phylogenetic distance (wU and uwU distances) of bacterial communities and the pairwise geographical distance of locations of each bark beetle using the Mantel test were not significant at the geographical space analyzed (_RMA_*r*_s_ = 0.07–0.11, *p*_s_ > 0.05, Table S3).

### 2.3. Prediction Roles of Gut Bacterial Communities

**Figure 6:**
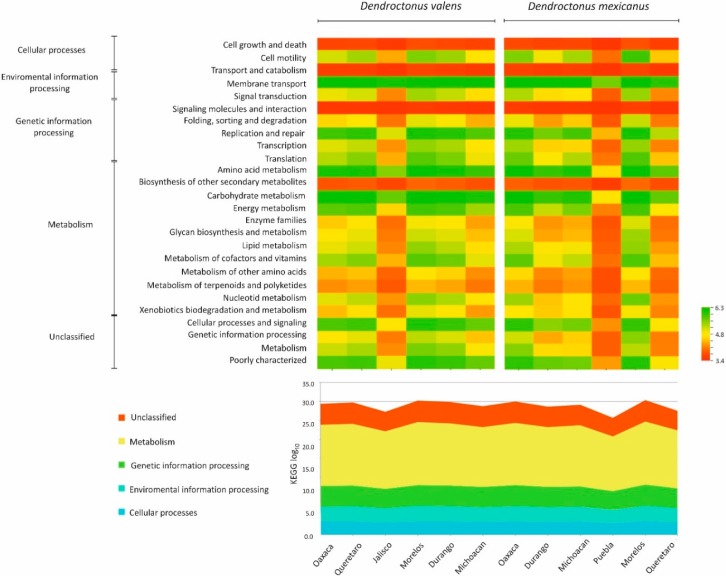
Heatmap of PICRUSt-inferred genes of bacterial communities of *Dendroctonus valens* and *D. mexicanus*. The range of colors indicates the relative abundance of metabolic pathways for each sample. Dark colors (green and yellow) indicate higher abundance and light (orange and red) ones lower abundance. The dendrogram shows the dissimilarities between the potential metabolic pathways of different bacterial communities clustered in KEGG 2 level. The relative abundance of the most important metabolic pathways in the KEGG 1 level on a log_10_ scale is shown below the heatmap figure.

Based on 79,349 and 92,660 reads for *Dendroctonus valens* and *D. mexicanus*, respectively, the predictive analyzes of the microbiota in the Kyoto Encyclopedia of Genes and Genomes (KEGG) pathways at level 2 (Figure 6) revealed a total of 195 metabolic pathways for gut bacterial communities for the former species and 203 for the latter. The Nearest Sequenced Taxon Index (NSTI) values varied from 0.020 to 0.031 (0.023 ± 0.003 standard deviation, SD) in *D*. *valens* locations, whereas these varied from 0.018 to 0.13 (0.064 ± 0.041 SD) in *D*. *mexicanus* locations, indicating accurate metabolic predictions of the bacterial metagenome of both bark beetles. The heatmap, based on the whole gut bacterial communities of each bark beetle, revealed the presence of genes putatively important for amino acid, carbohydrate, and vitamin metabolism (Figure 6).

The ANOSIM test did not show statistically significant differences among metabolic pathways of bacterial communities of *D*. *valens* (*R* = −0.18, *p* > 0.05) and *D*. *mexicanus* (*R* = −0.23, *p* > 0.05) across the geographical locations.

## 3. Discussion

One of the poorly studied aspects about the bacterial communities in insects, including bark beetles, is whether their structure (i.e., the composition and abundance of their members) and function (metabolic pathways) change across geographic space [41,42]. Our findings show that the gut bacterial community structure of bark beetles *Dendroctonus valens* and *D. mexicanus* is apparently stable across the geographical locations, as suggested by the values of richness and diversity indices that did not show significant differences among localities, except the Michoacan location in *D. valens*, whose geographical distance to its nearest pair (Jalisco) is 160.2 km and to its furthest pair (Oaxaca) the 608.2 km (Figure 1).

These findings agree with those reported for the microbial communities in the pine weevil *Hylobious abietis*, which are similar at lower taxonomic levels (family and genus) across locations in Europe [32], but they were different from those reported for *Dendroctonus valens* from different geographical sites in the United States of America [23]. Whereas it is difficult to recognize the factors that can drive these differences in *D. valens*, some of them may be the number of insects sampled, the anatomical site analyzed (whole insects, tree host, gut, exoskeleton, mycangium), the methodology employed (molecular cloning, DGGE or Next Generation Sequencing (NGS) methodologies), and the geographical scale studied.

The Venn diagram shows that the *Dendroctonus* species have an intraspecific core bacteriome different in genera number (Figure 4) and relative abundance (Figure 2). However, despite these differences, they share a minimal number of genera, among which stand out *Enterobacter*, *Pantoea*, *Providencia*, *Pseudomonas*, *Rahnella*, and *Serratia* (Figure 4). This small group of bacterial genera is the same as those reported for the *Dendroctonus* species [28], indicating that the intraspecific core bacteriome of these bark beetles, and perhaps of each *Dendroctonus* species, is higher than the interspecific core bacteriome reported for *Dendroctonus*. Based on these results, we hypothesize that each *Dendroctonus* species may have a specific core bacteriome, which can include all or almost all the members of the interspecific core bacteriome known to the *Dendroctonus* genus [28]. This closed group may support the basic metabolic functions of these bark beetles independent of the number of localities sampled and geographical space analyzed, as reported in other studies [32].

Our findings regarding β-diversity with unweighted UniFrac show that bacterial communities of both bark beetles vary geographically. The PCoA (uwU) and heatmap (Figure 3 and Figure 5b,d) show that this variation in both bark beetles is a result of the presence or absence of rare members with low relative abundance (<1% reads), as well as due to some dominant genera. In fact, some members of the intraspecific core bacteriome of these bark beetles are not necessarily the most abundant within the community, and their relative abundance varies among locations. This same pattern in the variation of β-diversity, given by the less-frequent members, has been observed in other studies carried out in other scolytines [3,26,27,28,32].

Our Mantel test results indicate that the spatial variation of gut bacterial communities in both bark beetles is not associated with the geographic distance, suggesting that the dominant genera (>1% relative abundance), including the strict core bacteriome members of both bark beetles, are present in all the geographical localities analyzed. Our findings agree with those reported for the pine weevil *Hylobius abietis* [32] but not with those reported for *Dendroctonus valens* at a wider geographical space [23]. This difference observed in *D. valens* with respect to geographical distance may be explained by the different geographical distances analyzed and the statistical coverage of the techniques used. In this study, we used NGS technologies and the maximum geographical distance between the sites was 1175 km, but Adams et al. [23] used the DGGE technique, and the maximum distance between their localities was 2500 km.

The factors that influence the structure to the bacterial communities should be determined in further studies. As suggested for other insects [43,44,45] and some *Dendroctonus* species [21,31,46], it is possible that geographical distance is not an important factor that influences these differences, particularly considering that pine-associated endophytic bacterial communities are highly similar and independent of their geographical location [38,47,48].

In addition, it is known that the gut is a micro-environmentally heterogeneous habitat and hence limiting for many bacteria, where the prevalence or not of specific bacteria within this system is not random [49]. In the case of bark beetles’ guts, different ecological and demographic factors (e.g., interactions, competition, population growth, resource availability) may determine the presence and/or dominance of bacterial groups and mutualistic relationships between them, at least between members of the bacteriome. Moreover, when the physiological conditions in the gut of bark beetles change, the pathogenic or commensal capacities of some bacteria may be expressed in this system [50].

Independent of the absence or presence of members at low frequency, the metabolic pathways inference analyzes with PICRUSt suggest that the basic biochemical functions of the microbiota and their contribution to the host biology are apparently conserved. In fact, whereas a considerable number of functional metabolic pathways were predicted for the gut bacterial communities of both the *Dendroctonus* species, the most important metabolic pathways were those related to nutrition and detoxification. Of these, carbohydrate metabolism, cofactors, and vitamins, as well as the biodegradation and metabolism of terpenoids and xenobiotic compounds were the most frequent. These functions are fundamental because these bark beetles feed on a substrate rich in organic compounds, non-essential amino acids, and structural carbohydrates, such as hemicellulose and cellulose, that are not easily degraded by insects. Additionally, throughout their entire life cycle, bark beetles are in contact with plant defensive compounds, some highly toxic, such as limonene and pinene, that can cause cellular damage and kill the *Dendroctonus* species [51,52,53]. Lastly, future studies using metatranscriptomics are necessary to confirm these inferences based on the 16S rRNA gene because to the best of our knowledge, there are no associative studies between the taxonomy assignments using this gene and specific functional pathways or genes in the *Dendroctonus* species, further, the results generated from this research using next-generation sequencing could expand knowledge for the control and management of these bark beetles.

## 4. Materials and Methods

### 4.1. Insect Collection, Dissection and DNA Extraction

Emerged adults of *D. valens* and *D. mexicanus* were directly collected from naturally infested pine trees in six distinct geographical locations in Mexico during 2017 (Figure 1, Table S1). The largest geographical distance between localities was 1175 km (Durango-Oaxaca sites), and the shortest distance was 160 km (Morelos-Puebla sites). Insects were removed from their galleries using fine sterile forceps, placed in sterile polycarbonate containers with wet paper to avoid insect desiccation, stored at 4 °C for their transport, and processed immediately after arriving at the laboratory.

Two sets of 30 insects each were taken in each locality, to integrate two biological replicates for each *Dendroctonus* species. The insects were dissected under sterile conditions as described by Briones-Roblero et al. [9]. The last washing water was inoculated in Petri dishes with trypticase soy agar (TSA, BD, Difco, Sparks, MD, USA) inoculated with the last rinsing water and by negative Polymerase Chain Reaction (PCR) amplification of the same water to assess the efficiency of the disinfestation. The plates were incubated at 28 °C for 48–72 h. Each set of 30 guts was processed independently for DNA extraction with DNeasy Blood and Tissue Kit (Qiagen, Valencia, CA, USA) according to the manufacturer’s protocol.

### 4.2. Bacterial 16S rRNA Polymerase Chain Reaction Amplification and Pyrosequencing

The V1–V3 region of the 16S rRNA gene was amplified using universal primers 8F and 556R [54], fitted to 10 bp multiplex identifiers (MID) and Roche 454 adaptors for the Lib-L protocol. PCR reactions were performed in a thermal cycler Techne TC 5000 (Techne, Staffordshire, UK) in a final volume of 25 µL containing 80 ng DNA template, 1× reaction buffer, 2.0 mM MgCl_2_, 0.4 pM of each primer, 0.4 mM of each dNTPs, and 1.0 U of Platinum Taq DNA polymerase High Fidelity (Invitrogen™ Life Sciences, Carlsbad, CA, USA). The reaction conditions were as follows: initial denaturation at 94 °C for 5 min, 25 cycles denaturation at 94 °C for 50 s, annealing temperature 53 °C for 50 s and extension at 72 °C for 50 s, and a final extension 72 °C for 5 min.

The amplification products of three individual PCR reactions for each biological replicate were combined and purified using a QIAquick Gel Extraction kit (Qiagen, Valencia, CA, USA). The amplicons of all samples were pooled in equal volumes for pyrosequencing using a Roche GS-FLX Titanium 454 pyrosequencer (Roche, Mannheim, Germany) in Macrogen Inc. (Seoul, Korea).

### 4.3. Data Pyrosequencing Analysis

The data analysis was conducted using the software Quantitative Insight into Microbial Ecology (QIIME) v 1.9 (available online: http://qiime.org) [55]. All low-quality reads (Phred quality score < 25) and sequences <200 or >550 bp long containing ambiguous characters, homopolymers > 6 bp, and mismatches in primers > 1 were removed from subsequent analyzes [56].

The high-quality sequences were grouped in OTUs through the open-reference picking method at a 97% similarity threshold [57] using Uclust v 1.2.22 [58]. Chimeric sequences were identified with Chimera Slayer v 1.39.5.0 [59] and then removed from the data set. The most abundant read of each OTU was selected as a representative sequence and then aligned with the Greengenes core sequences set (available online: http://greengenes.lbl.gov/) using PyNast [60].

The taxonomic assignment from phylum to genus for representative sequences was done at a ≥ 0.8 confidence score with the Ribosomal Database Project (RDP) Classifier (available online: https://rdp.cme.msu.edu/index.jsp) to know its taxonomic identity [61]. The taxonomic assignment was manually corroborated by comparing with the closest matched sequences on three databases (RDP, GenBank, and Greengenes), the cutoff being from 97% to 100% at the genus level. A Maximum-Likelihood (ML) phylogenetic inference analysis was performed in PhyML (available online: http://atgc.lirmm.fr/phyml/), using only representative sequences at the genus level. To this analysis, these sequences were aligned in Clustal X v 2.0.10 (available online: http://www.clustal.org/clustal2/) [62] and trimmed at their 5′ and 3′ ends in SeaView v 4 (available online: ftp://pbil.univ-lyon1.fr/pub/mol_phylogeny/seaview/archive/). The best-fit model of nucleotide substitution was selected with JModeltest v 2.1.7 (available online: http://darwin.uvigo.es/our-software/) [63] based on the Akaike Information Criterion (AIC). The reliability of each node was estimated via a bootstrap analysis after 1000 pseudoreplicates. Finally, the phylogeny and heatmap of relative abundances were matched using Interactive Tree of Life (iTol) (available online: https://itol.embl.de/) [64].

### 4.4. Bacterial Diversity Analysis

As the amount of sequences analyzed could affect the diversity analysis, we used rarefied data running the multiple rarefaction script implemented in QIIME v 1.9 (for each sample, we calculated the number of OTUs expected to be observed for 100 reads). To determine the probability that a randomly selected amplicon from a sample was previously sequenced, the Good’s coverage was calculated as an estimator of sampling completeness [65]. Estimators of richness (Chao1), diversity (Simpson and Shannon), and phylogenetic diversity (PD) were used to determine the α-diversity in bacterial communities for each bark beetle species and biological replicates [66,67]. The normality and homogeneity of the variances of these estimators were tested with a Shapiro-Wilkinson test and *F* test [68]. As the diversity indices did not meet the assumptions of equal variances, those values were compared using Welch’s *F* test for ANOVAs and its respective post hoc multiple paired comparisons using Dunn’s test [69].

The β-diversity of gut bacterial communities among locations was estimated using unweighted (considers only phylogenetic richness) and weighted (considers both relative abundance and phylogenetic richness) Fast UniFrac distances [70], as well as the Bray-Curtis dissimilarity index [71]. The Monte Carlo method was performed to test the statistically significant differences among the bacterial communities using both Fast UniFrac distances [70], while the Adonis test was used to evaluate the differences with Bray-Curtis distances. A Principal Coordinate Analysis (PCoA) using unweighted and weighted UniFrac distances was performed in NTSYS-PC v 2.02 (Exeter Software, Setauket, New York, NY, USA) to explore multidimensional patterns of diversity variation of bacterial communities among locations [72].

### 4.5. Core Bacteriome

The strict core bacteriome of these bark beetles was determined according to Hernández-García et al. [28]. We, only include those bacterial genera present in all localities and their replicates, whose relative frequencies were >1%. To identify unique and shared OTUs among localities of both the *Dendroctonus* species, a Venn diagram was generated in Bioinformatics and Evolutionary Genomics (available online: http://bioinformatics.psb.ugent.be/webtools/Venn/).

To determine the variation of bacterial communities of each bark beetle with respect to geographical distance, a nonparametric Mantel test was used to determine the correlation between the matrix of pairwise geographic distance among localities and pairwise phylogenetic distance matrix, using both weighted and unweighted UniFrac [73]. The randomized matrices were generated by row and column permutation. The randomization was performed 5000 times to establish a 95% confidence interval [73]. However, given that the estimation and measurement of the correlation test between the geographic and phylogenetic distance matrices of bacterial communities were not free of errors, it was necessary to estimate the slope and *y*-intercept to find the best straight line that described this association in a confinable way. To do this, we used the reduced major axis (RMA) method and 95% confidence intervals for elements of the line that were calculated using a bootstrap test as implemented on PAST v 3.20 (available online: https://folk.uio.no/ohammer/past/) [69].

### 4.6. Predictive Functional Analysis Based on Metagenomic 16S rRNA Surveys

The predicted functional profiles of bacterial communities associated with the gut of *D. valens* and *D. mexicanus* of each locality were inferred with the bioinformatic tool PICRUSt [74]. The sequences from each library were demultiplexed and used to generate the OTU table in BIOM format, following a closed-reference method. Given that the ability of PICRUSt to estimate a metabolic profile relies on a set of known sequenced genomes, the OTU table was normalized and used to make a comparison with sequenced bacterial genomes deposited in the Kyoto Encyclopedia of Genes and Genomes (KEGG) [75] at hierarchical levels 2 and 3.

The obtained tables with the counts of predicted genes per sample were cleaned according to the following criteria: Removal of categories unrelated to bacterial physiology/metabolism (similar to human diseases) and removal of gene family categories with a count equal to zero. The functional predictions were plotted at the hierarchical levels 2 and 3 of KEGG. To evaluate the prediction accuracy of samples, we calculated the Nearest Sequenced Taxon Index (NSTI). The NSTI scores summarize the extent to which microorganisms in a sample are related to sequences’ genomes, and they represent the average branch length that separates each OTU from a reference bacterial genome, weighting their relative abundance in each sample [74]. Lastly, to compare whether the metabolic pathways among the gut bacterial communities of different locations of both the *Dendroctonus* species were different, we performed an analysis of similarity (ANOSIM) using a Gower index with 10,000 permutations in PAST v 3.20 [69].

### 4.7. Data Accessibility

The pyrosequencing-derived 16S rRNA gene sequence datasets were submitted to the NCBI database, under accession number of Sequence Read Archive (SRA) SRP158467.

## Figures and Tables

**Table 1:** Summary of pyrosequencing data and alpha diversity of *Dendroctonus valens* and *D. mexicanus.*

Label	No. of High Quality Reads	Observed OTUs^97^	Richness and Diversity Index					Good’s Coverage
			Chao1	PD *	Shannon	Simpson	Simpson^−1^	
***D. valens***								
Oaxaca I	8300	118	232	5.20	3.6	0.866	7.5	0.973
Oaxaca II	2703	115	234	4.73	3.7	0.872	7.8	0.974
Queretaro I	6403	173	371	5.64	4.7	0.930	14.3	0.960
Queretaro II	13,305	158	364	5.79	4.9	0.944	17.8	0.965
Morelos I	11,589	174	390	6.51	4.6	0.916	11.9	0.961
Morelos II	5815	153	327	6.50	4.2	0.896	9.6	0.966
Jalisco I	13,459	87	118	3.11	3.6	0.851	6.7	0.986
Jalisco II	3713	164	314	4.94	4.5	0.899	9.9	0.965
Durango I	8409	109	224	4.68	3.4	0.834	6.0	0.975
Durango II	4723	158	251	5.41	4.2	0.898	9.8	0.968
Michoacan I	3461	100	268	4.13	3.4	0.859	7.1	0.975
Michoacan II	4469	117	196	5.59	3.5	0.861	7.2	0.975
***D. mexicanus***								
Oaxaca I	7310	95	143	3.68	4.0	0.899	9.9	0.981
Oaxaca II	6836	60	95	2.27	2.5	0.748	4.0	0.988
Queretaro I	4436	91	166	3.19	2.8	0.649	2.8	0.981
Queretaro II	3589	73	104	1.95	3.3	0.789	4.7	0.989
Morelos I	37,466	73	184	2.82	2.5	0.623	2.7	0.982
Morelos II	4444	95	163	4.47	3.7	0.828	5.8	0.980
Puebla I	4681	87	120	2.91	3.0	0.741	3.9	0.983
Puebla II	2906	72	89	2.66	3.0	0.743	3.9	0.989
Durango I	16,022	82	139	2.95	3.0	0.775	4.4	0.984
Durango II	5786	65	83	2.10	3.2	0.798	4.9	0.990
Michoacan I	3589	102	154	3.42	2.9	0.641	2.8	0.980
Michoacan II	4447	97	125	4.14	3.5	0.770	4.3	0.985

* PD: Phylogenetic Diversity.

## Supplementary Materials

Supplementary Materials can be found at http://www.mdpi.com/1422-0067/19/9/2578/s1.

## Abbreviations

AIC	Akaike Information Criterion
ANOVA	Analysis of Variance
DGGE	Denaturing Gradient Gel Electrophoresis
KEGG	Kyoto Encyclopedia of Genes and Genomes
NGS	Next Generation Sequencing
NSTI	Nearest Sequenced Taxon Index
NTSYS-PC	Numerical Taxonomy and Multivariate Analysis
OTU	Operational Taxonomic Unit
PCoA	Principal Coordinate Analysis
PD	Phylogenetic Diversity
PICRUSt	Phylogenetic Investigation of Communities by Reconstruction of Unobserved States
QIIME	Quantitative Insight into Microbial Ecology
RDP	Ribosomal Database Project
uwU	Unweighted UniFrac
wU	Weighted UniFrac
